# Growth inhibition of androgen-responsive prostate cancer cells with brefeldin A targeting cell cycle and androgen receptor

**DOI:** 10.1186/1423-0127-17-5

**Published:** 2010-01-26

**Authors:** Srinivas Rajamahanty, Catherine Alonzo, Shahrad Aynehchi, Muhammad Choudhury, Sensuke Konno

**Affiliations:** 1 Department of Urology, New York Medical College, Valhalla, NY, USA

## Abstract

**Background:**

Androgen ablation is one of the viable therapeutic options for patients with primary hormone (androgen)-dependent prostate cancer. However, an antibiotic brefeldin A (BFA) has been shown to exhibit the growth inhibitory effect on human cancer cells. We thus investigated if BFA might inhibit proliferation of androgen-responsive prostate cancer LNCaP cells and also explored how it would be carried out, focusing on cell cycle and androgen receptor (AR).

**Methods:**

Androgen-mediated cellular events in LNCaP cells were induced using 5α-dihydrotestosterone (DHT) as an androgenic mediator. Effects of BFA on non-DHT-stimulated or DHT-stimulated cell growth were assessed. Its growth inhibitory mechanism(s) was further explored; performing cell cycle analysis on a flow cytometer, assessing AR activity by AR binding assay, and analyzing AR protein expression using Western blot analysis.

**Results:**

DHT (1 nM) was capable of stimulating LNCaP cell growth by ~40% greater than non-stimulated controls, whereas BFA (30 ng/ml) completely inhibited such DHT-stimulated proliferation. Cell cycle analysis showed that this BFA-induced growth inhibition was associated with a ~75% reduction in the cell number in the S phase and a concomitant increase in the G_1 _cell number, indicating a G_1 _cell cycle arrest. This was further confirmed by the modulations of specific cell cycle regulators (CDK2, CDK4, cyclin D_1_, and p21^WAF1^), revealed by Western blots. In addition, the growth inhibition induced by BFA was accompanied by a profound (~90%) loss in AR activity, which would be presumably attributed to the significantly reduced cellular AR protein level.

**Conclusions:**

This study demonstrates that BFA has a potent growth inhibitory activity, capable of completely inhibiting DHT (androgen)-stimulated LNCaP proliferation. Such inhibitory action of BFA appears to target cell cycle and AR: BFA led to a G_1 _cell cycle arrest and the down-regulation of AR activity/expression, possibly accounting for its primary growth inhibitory mechanism. Thus, it is conceivable that BFA may provide a more effective therapeutic modality for patients with hormone-dependent prostate cancer.

## Background

Although androgens are essential for the development and growth of normal prostate, they are also responsible for the development of benign prostatic hyperplasia and prostate cancer [1]. Androgen ablation therapy is a viable treatment modality for patients with primary hormone (androgen)-dependent prostate cancer, lowering the serum androgen level and blocking androgen receptor (AR)-mediated signal transduction [2,3]. AR is a member of the steroid/nuclear receptor super family [1,3] and its major biological role has been well documented. Androgen binds to AR to form the androgen-AR complex that is required for nuclear translocation, followed by its binding to the androgen-responsive element (ARE) for transcriptional activation of androgen-responsive genes including prostate-specific antigen (PSA) [4]. PSA is thus under the androgenic control and currently the most commonly used biomarker for the diagnosis and prognosis of prostate cancer, by measuring the level/amount of serum PSA (secreted from prostate epithelial cells) [5]. After all, AR is the primary factor transmitting an androgenic signal to the nucleus for proliferation of prostate (cancer) cells as well as the regulation of androgen-mediated cellular events.

Antiandrogens [2,6] such as cyproterone acetate, nilutamide, flutamide, bicalutamide etc. are then used to abolish androgenic effects on prostate cancer cells, by competing with androgen for AR binding to consequently slow down or inhibit their growth. In addition, luteinizing hormone-releasing hormone (LHRH) agonists (e.g., leuprolide, goserelin, triptorelin etc.) [7] are also used to reduce availability of circulating androgens to cancer cells by suppressing testicular steroidogenesis (i.e. testosterone synthesis). In some cases, the combinations of antiandrogens and LHRH agonists are given to patients to improve treatment efficacy; however, the overall efficacy of these trials has been shown to be rather low with limited duration, resulting in an almost inevitable cancer progression [3]. This led us to assume that besides blocking the AR or manipulating the androgen level, there must be a more effective modality for managing hormone-dependent prostate cancer. We then explored certain drugs/agents that directly and specifically interfere with the androgen-mediated growth pathway in prostate cancer.

Brefeldin A (BFA) [8], a fungal antibiotic, has been initially known to play a regulatory role in the intracellular transport system [9-11]. It induces the reversible disassembly of the Golgi complex, resulting in the interruption of protein transport from the endoplasmic reticulum (ER) to the Golgi [9,10]. BFA has been also shown to collapse the Golgi complex into the ER, redistributing Golgi-associated proteins/enzymes to the ER [11]. In addition, BFA has other biological properties such as antitumor, antiviral, antifungal, and antimitotic effects [10]. Particularly, BFA-induced apoptosis and growth inhibition have been shown in several human cancer cells, including leukemia, colon, prostate (androgen-independent), and primary prostatic adenocarcinoma cells [12-16]. Moreover, an *in vitro *screen of human tumor cells conducted by the National Cancer Institute (USA) also confirmed that BFA had a potent antiproliferative activity on several strains of prostate cancer cells [17].

Accordingly, we investigated the potential effect of BFA on androgen-mediated prostate cancer cell growth, focusing on the cell cycle [18] and AR regulation in androgen-responsive prostate cancer LNCaP cells [19]. BFA was then found to have a potent growth inhibitory activity, through a blockage of the cell cycle progression and the down-regulation of AR activity and expression. More details are described herein.

## Methods

### Cell culture

The androgen-responsive human prostatic cancer LNCaP cells were obtained from the American Type Culture Collection (Rockville, MD) and grown in RPMI 1640 medium supplemented with 10% fetal bovine serum (FBS) and penicillin/streptomycin (100 units/ml and 100 μg/ml). They were maintained at 37°C in a humidified incubator in an atmosphere of 95% air and 5% CO_2_. The medium was routinely changed every 3 days and cells were passaged or split weekly with trypsinization. For experiments, androgen-mediated cellular responses in LNCaP cells were studied using the medium containing 5% charcoal-stripped fetal bovine serum (CS-FBS) [20] in place of FBS, in which endogenous steroids were removed to allow cells to primarily respond to exogenously added androgens. Cells (2 × 10^5 ^cells/ml) were seeded in 6-well plates (2 ml/well) or T-75 flasks (10 ml/flask) and treated with brefeldin A (BFA) (Epicentre Technologies, Madison, WI), DHT (5α-dihydrotestosterone) (NEN-DuPont, Boston, MA), or BFA/DHT combination. As a stock BFA was prepared in ethanol, a vehicle culture was also set up by adding the same amount of ethanol used in BFA to the cells. Cell numbers were then determined at specified times by cell count using the trypan blue exclusion method. All chemicals and reagents used in this study have the great purity of at least >95%.

### Cell cycle analysis

Cell cycle phase distributions were determined on a FACScan flow cytometer (Becton-Dickinson) equipped with a double discrimination module. Control or agents-treated cells (~1 × 10^6 ^cells per condition) were harvested, washed twice with phosphate-buffered saline (PBS), and resuspended in 500 μl of propidium iodide solution (20 μg/ml propidium iodide, 0.2 mg/ml RNase, 0.2 mg/ml EDTA, 0.5% Nonidet P-40) for 1-h incubation at room temperature in the dark. Following incubation, ~10,000 nuclei from each sample were analyzed on a flow cytometer, and CellFit software was used to quantify cell cycle compartments to estimate the % of cells distributed in the different cell cycle phases.

### Western blot analysis

The procedures essentially followed the protocol described elsewhere [16]. Cell pellets from control or agents-treated cells were resuspended in cell lysis buffer (10mM HEPES-KOH, pH 7.5, 90mM KCl, 1.5mM Mg(OAc)_2_, 5% glycerol, 0.5% NP-40, 1mM DTT and 1mM PMSF). Cells lysates were then prepared by freezing-thawing three times in liquid nitrogen and their protein concentrations were determined using Coomassie protein assay reagent (Pierce, Rockford, IL) on a spectrophotometer. An equal amount of cell lysates (10 μg) was first subjected to 10% SDS-PAGE (polyacrylamide gel electrophoresis), followed by protein transfer to a nitrocellulose membrane with a semidry electroblotter apparatus (MilliBlot, Millipore). After an overnight blocking of membrane with 3% non-fat milk in TBST (20mM Tris-HCl, pH 7.6, 137mM NaCl, 0.05% Tween-20), the blot was incubated for 90 min with the primary antibodies against CDK2, CDK4, cyclin D_1_, p21^WAF1 ^(all purchased from Santa Cruz Biotechnology, Santa Cruz, CA) or AR (anti-AR from Affinity BioReagents, Golden, CO), followed by a 30-min incubation with the appropriate secondary antibodies conjugated with peroxidase. After washing the blot with PBS, specific immunoreactive proteins were demonstrated by chemiluminescence, following the manufacturer's protocol (Kirkegaard and Perry Laboratories, Gaithersburg, MD). The detected protein bands on the X-ray film (autoradiogram) were then quantified using scan densitometry (Silk Scientific, Oregon, UT).

### Androgen receptor (AR) binding assay

The protocol was adopted from the method of Turcotte et al. [21] with minor modifications. Radioactive methyltrienolone or R1881, the synthetic analog of DHT, was used as a ligand for AR. After LNCaP cells were cultured with BFA (30 ng/ml) for indicated times, they were washed with a plain medium and incubated with 10 nM [^3^H]-R1881 (87 Ci/mmol, NEN-DuPont) for 2 h at 37°C in the presence or absence of 100-fold excess of unlabeled DHT. Following incubation, cells were washed extensively with PBS to remove unbound radioactive ligand and then solubilized in 0.3N NaOH-ethanol (4:1; v/v) for 5 min. Those solubilized cells were transferred to the scintillation vials for measuring the radioactivity incorporated into AR (i.e. a ligand-AR binding) using a scintillation counter. Specific AR-binding was then normalized by subtracting non-specific binding (background) and expressed by cpm/10^6 ^cells.

### Tandem PSA assay

Cell lysates were prepared from control and BFA-treated cells by freezing-thawing in liquid nitrogen as described earlier. Either 100 μl aliquots of spent media or 10 μg of cell lysates were used for Tandem-E PSA assay (Hybritech, San Diego, CA). The quantitative measurement of PSA was performed by following the manufacturer's assay procedures.

### Statistical analysis

All data were presented as mean ± SD (standard deviation) and statistical differences between the groups were assessed with the unpaired Student's *t *test. A value of *p *< 0.05 was considered significant.

## Results

### Effect of BFA on cell growth

**Figure 1:**
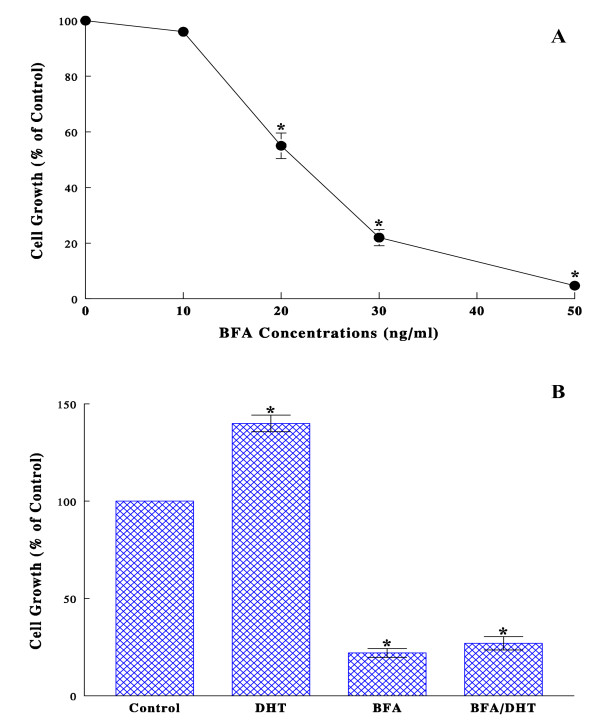
**Effects of BFA on LNCaP cell growth**. (A) Dose-dependent effects of BFA on LNCaP cell growth were assessed: after cells were cultured with 0-50 ng/ml of BFA for 72 h, those viable cell numbers were determined and expressed by the percent (%) relative to cell number in control (100%). (B) Effects of BFA on DHT-stimulated cell growth were examined: cells were grown with DHT (1 nM), BFA (30 ng/ml), or their combination for 72 h, and cell growth was assessed by the % of controls. All data are mean ± SD (standard deviation) from three separate experiments (*p < 0.05 compared with controls).

LNCaP cells were first cultured with the varying concentrations (0-50 ng/ml) of BFA for 72 h, and their effects on cell growth were assessed by cell count. Cell growth was significantly inhibited by BFA in a dose-dependent manner, resulting in a 45, 78, and 95% growth reduction with 20, 30, and 50 ng/ml of BFA, respectively (Fig. 1A). The moderately effective BFA concentration of 30 ng/ml, which appeared to be adequate for our purpose, was then used in the rest of the studies. The cell viability test also indicated that the growth inhibition induced by BFA (30 ng/ml) was unlikely due to cell death, confirming an ~80% cell viability. Since LNCaP cell proliferation is known to be regulated by androgens [19], the effect of BFA on such an androgen-responsive cell growth was next examined by culturing the cells with DHT (1 nM), BFA (30 ng/ml), or BFA/DHT combination in CS-FBS medium [20] for 72 h. As expected, DHT indeed led to a ~40% greater cell growth (than controls) but this stimulated growth was completely inhibited by BFA, as evidenced by a drastic growth reduction with the BFA/DHT combination (Fig. 1B). Thus, these results suggest that BFA could be a potent growth inhibitor, interfering with the DHT-mediated growth pathway in LNCaP cells.

### Effects of BFA on cell cycle

**Figure 2:**
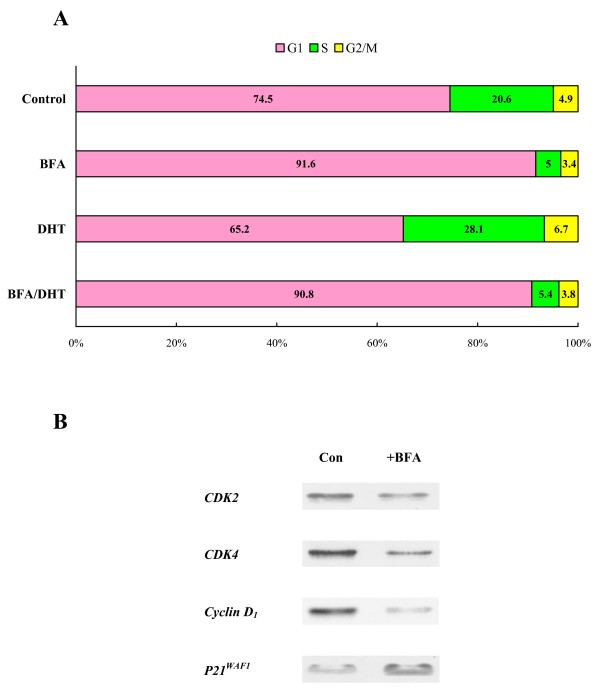
**Effects of BFA on cell cycle**. (A) LNCaP cells were treated with DHT (1 nM), BFA (30 ng/ml), or BFA/DHT combination for 72 h and cell cycle analysis was performed as described in Methods. Cell cycle phase distributions or the number (%) of cells present at the G_1_, S, or G_2_/M phases in each experimental condition was determined and plotted. The data were mean ± SD from three separate experiments and subjected to statistical analysis; however, only those mean values (without SD) were used for plotting the graph for a clear illustration. (B) After cells were treated with or without BFA (30 ng/ml) for 72 h, the expressions of specific cell cycle regulators such as CDK2, CDK4, cyclin D_1_, and p21^WAF1 ^were analyzed on Western blots and autoradiographs of those regulators (on the X-ray film) are shown.

To explore the underlying mechanism of BFA-induced growth suppression, cell cycle analysis was performed on the cells that had been treated with DHT (1 nM), BFA (30 ng/ml), or BFA/DHT combination (in CS-FBS medium) for 72 h. Compared with control cells, a >35% increase (*p *< 0.05) in the S phase cell number (while a ~13% decrease in the G_1 _cell number) was observed in DHT-treated cells (Fig. 2A), consistent with the accelerated growth rate (Fig. 1B). In contrast, (compared with controls) a ~75% decrease (*p *< 0.01) in the S phase cell number, concomitant with a ~20% increase (*p *< 0.05) in the G_1 _cell number, was seen with sole BFA as well as BFA/DHT treatments (Fig. 2A), resulting in a significant growth reduction (Fig. 1B). These results thus suggest that BFA appears to cause a blockage of cells entering from the G_1 _to the S phase, i.e. a G_1 _cell cycle arrest, ultimately leading to the growth cessation.

For confirmation, the effects of BFA were also examined on the specific cell cycle regulators for the G_1_-S phase transition [18], including CDK2, CDK4, cyclin D_1_, and p21^WAF1^. Cells treated with or without BFA (30 ng/ml) for 72 h were subjected to Western blot analysis, followed by densitometric quantification. The expressions of CDK2, CDK4, and cyclin D_1 _were significantly (>60%) reduced or down-regulated (compared with controls), while p21^WAF1 ^expression was up-regulated in BFA-treated cells (Fig. 2B). This further supports the notion that BFA-induced growth inhibition is associated with a G_1 _cell cycle arrest.

### Effects of BFA on androgen receptor (AR) activity and expression

**Figure 3:**
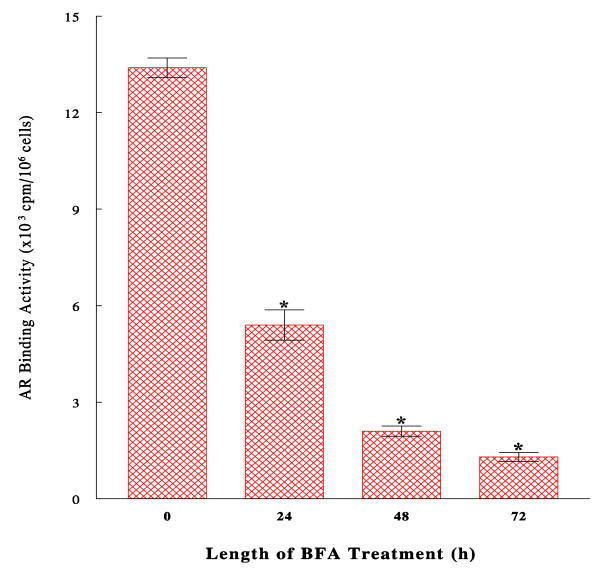
**Effects of BFA on androgen receptor (AR) binding activity**. Cells were cultured with BFA (30 ng/ml) for 24, 48, or 72 h, and AR binding assays were performed as described in Methods. Specific AR activity was calculated and expressed by cpm incorporated/10^6 ^cells. The data are mean ± SD from three independent experiments (*p < 0.03 compared with controls at 0 h).

Since AR is a major factor playing an essential role in the androgen-dependent prostate cancer growth [1], we then examined the effects of BFA on (biological) activity and expression of AR. First, AR activity was assessed by AR binding assay, measuring the binding potential of AR to DHT, in the cells treated with BFA (30 ng/ml) for 24, 48, and 72 h. Such studies showed that AR binding activity was significantly (~60%) decreased by 24-h BFA treatment and further diminished by ~90% after 72 h (Fig. 3), indicating a time-dependent, progressive loss in AR activity by BFA.

**Figure 4:**
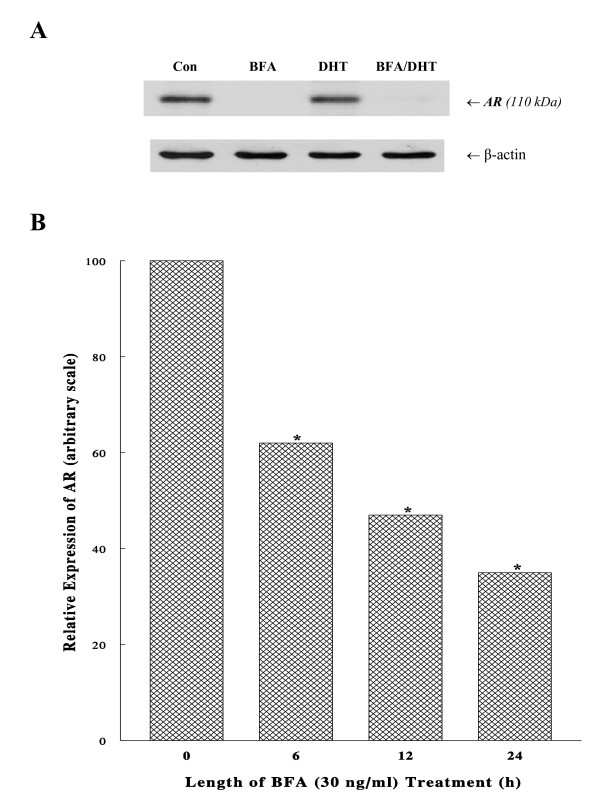
**Effects of BFA on AR expression**. (A) After cells were cultured with BFA (30 ng/ml), DHT (1 nM), or BFA/DHT combination for 72 h, cell lysates (10 μg) were prepared and analyzed for AR expression using Western blots and autoradiograph of those AR protein bands (110 kDa) is shown. Beta (β)-actin is also shown as a protein loading control. (B) Following the exposure of cells to BFA (30 ng/ml) for 0, 6, 12, or 24 h, the time-dependent reduction in AR expression was analyzed on Western blots. Intensities of AR expressions detected on autoradiograph were quantified using a scan densitometer and expressed by the arbitrary values (*p < 0.05) relative to controls (at 0 h) normalized to 100. The data are mean ± SD from three independent experiments.

To better understand such a profound reduction in AR activity, the cellular status (expression) of AR protein following DHT, BFA, or BFA/DHT treatments (in CS-FBS medium) for 72 h was analyzed using Western blots. Although AR expressions in both control and DHT-treated cells were similarly high and apparent, BFA remarkably down-regulated its expression by > 90% (Fig. 4A). This study also revealed that DHT was unable to neutralize or prevent BFA-induced AR diminution or BFA simply overcame androgenic effect of DHT on AR integrity, resulting in only a marginal level of AR detected in BFA/DHT-treated cells (Fig. 4A).

Moreover, a nearly complete disappearance or degradation of AR with BFA led us to assume that BFA might have primarily targeted AR at the early time point. Such a possibility was then tested by treating the cells with BFA (30 ng/ml) for 6, 12, or 24 h, and AR expressions were analyzed on Western blots and quantified with a scan densitometer. The results showed that the AR level decreased to ~60% (p < 0.05) of the initial level after only 6-h BFA treatment and steadily declined to 47% and 35% (p < 0.05) at 12 h and 24 h, respectively (Fig. 4B). Taken together, these studies demonstrate that BFA is capable of progressively down-regulating AR expression, presumably accounting for a drastic (~90%) loss in its binding (biological) activity at 72 h (Fig. 3).

### Effect of BFA on androgen-regulated PSA secretion

**Figure 5:**
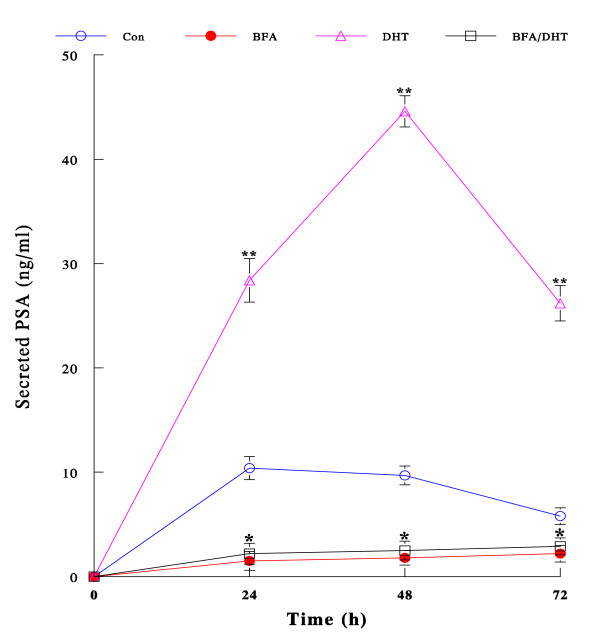
**Effect of BFA on PSA secretion regulated by DHT**. Cells were cultured with BFA (30 ng/ml), DHT (1 nM), or BFA/DHT combination for 24, 48, and 72 h. Spent media were collected at each time point and assayed for secreted PSA. The amount of PSA secreted was expressed by ng/ml and all data are mean ± SD from three independent experiments (*p < 0.05; **p < 0.005 compared with controls).

As BFA appears to profoundly down-regulate AR expression, it was of interest to examine whether BFA might also affect other androgen-mediated cellular events (via AR) such as secretion of PSA, which is a useful biomarker for prostate cancer and under the androgenic control [5]. LNCaP cells were cultured with BFA (30 ng/ml), DHT (1 nM), or BFA/DHT combination (in CS-FBS medium) for 72 h. Spent media were collected and assayed for the amount of PSA secreted (to culture media) at the indicated times. Time-dependent changes in secreted PSA are shown in Fig. 5. Control cells secreted the measurable amount of PSA although its levels seemed to gradually decline to 72 h. However, BFA notably (*p *< 0.05 compared with controls) inhibited PSA secretion throughout the experiments. In contrast, DHT dramatically accelerated PSA secretion up to a maximal 4.6-fold (*p *< 0.001 compared with controls) at 48 h and then its level considerably plunged by 72 h (although it was yet significantly higher than controls). However, this stimulatory effect of DHT (on PSA secretion) was almost completely abolished by BFA, bringing its PSA levels down to nearly the same as those in BFA alone. Thus, these results illustrate that BFA is capable of inhibiting PSA secretion, one of androgen-regulated cellular events, presumably through abolishing DHT stimulatory action due to AR degradation (down-regulation of AR expression) induced by BFA.

## Discussion

In the present study, we examined the growth inhibitory effect of BFA and also explored its inhibitory mechanism on the androgen-responsive human prostate cancer LNCaP cells *in vitro*. BFA demonstrated a potent growth inhibitory activity on these cancer cells and completely suppressed DHT-stimulated cell growth as well (Fig. 1B). This indicates that BFA appears to interfere with the DHT-mediated growth pathway in LNCaP cells. To have an insight into the mode of BFA action against DHT, two parameters, namely the cell cycle and the androgen receptor (AR), were subjected to further investigations. Cell cycle analysis revealed that BFA by itself caused a ~75% reduction in the S phase cell number, indicating a typical G_1 _cell cycle arrest. Similarly, BFA even blocked the DHT-stimulated G_1_-S phase progression. This BFA-induced G_1 _arrest was further supported by the down-regulation of CDK2, CDK4, and cyclin D_1 _while the up-regulation of p21^WAF1^, which were all known to play the key roles in the G_1_-S phase transition [18]. Thus, a cell cycle arrest in the G_1 _phase may at least in part account for the BFA-induced growth inhibition in LNCaP cells.

As AR is known to play a major role in the DHT-mediated prostate cancer growth [3], possible effects of BFA were examined on biological activity and cellular expression of AR. Such studies showed that AR activity and expression were both extensively (>90%) reduced following 72-h BFA treatment; particularly AR expression/protein has declined to 60% in merely 6-h of BFA treatment (Fig. 4B). Moreover, DHT failed to reverse or prevent antagonistic action of BFA on AR; in fact, BFA even severely diminished AR expression in DHT-stimulated cells to <*10% *(compare DHT with BFA/DHT in Fig. 4A). Thus, regardless of the presence of DHT, BFA appears to abrogate the cellular expression of AR. This BFA-mediated AR down-regulation was also verified by the time-dependent reduction in AR expression (Fig. 4B). In conjunction with a cell cycle arrest, these findings suggest that the BFA-induced inhibition of DHT-stimulated LNCaP proliferation also results from a loss of AR activity due to a diminution of AR protein.

Although DHT, acting through the AR, is essential for the growth of prostate cancer, the mechanism of androgen-stimulated cell proliferation has not been fully defined. For example, the signaling pathways responsible for the androgenic effects have been extensively studied [22], but the actual roles of such androgen-regulated genes/proteins in the mitogenic activity yet remain elusive. Meanwhile, the importance of the cell cycle regulation has been reported in the androgen-dependent proliferation of prostate cancer cells [23-25]. Mitogenic signals of androgen were shown to be mediated through multiple G_1 _regulatory elements/factors controlling the G_1_-S phase transition [23]. In fact, the down-regulated expressions of cyclin D_1_, CDK2, and CDK4 while the up-regulated p21^WAF1 ^were associated with a G_1 _growth arrest in LNCaP cells [24,25]. This finding was also consistent with BFA-induced G_1 _cell cycle arrest in our study (Fig. 2B). It is thus conceivable that a disruption of androgen (via AR)-stimulated cell cycle progression by BFA could be the vital mechanism of such a BFA-induced growth inhibition.

In addition, the finding that BFA was capable of severely down-regulating activity and expression of AR is rather significant, and this may deserve further discussion to understand and speculate exactly *how *BFA would carry it out to fully block androgenic action. It is yet uncertain at present whether the down-regulation of AR expression or AR degradation may take place primarily at the transcriptional or translational level. However, such BFA-induced AR degradation might be likely due to the *instability *of newly synthesized AR protein that failed to undergo the obligatory post-translational modification. As AR is a phosphoprotein, it needs to be post-translationally modified or *phosphorylated *in the Golgi complex to become matured and functional [26]. It is yet possible that BFA could inhibit such AR protein modification or maturation by blocking its transport from the ER to the Golgi complex [8,9]. Due to this BFA-interrupted AR phosphorylation, it is then plausible that unmodified AR protein would become rather unstable and susceptible to proteolysis, thereby being rapidly degraded. This is one possible notion that may account for how the AR protein level could be drastically reduced by BFA, or rather, by BFA-activated protease. In fact, we have been also exploring such protease(s) that would specifically target AR protein. Although we have not yet completed the study, the currently available data thus far indicate that "proteasome" [27], a multicatalytic protease, appears to be a primary candidate for targeting AR. We performed several studies using the specific inhibitors for proteases and proteasome to prevent BFA-induced AR degradation. We then found that only the proteasome inhibitor, clasto-lactacystin β-lactone (β-lac) [28], not other protease inhibitors, was nearly completely capable of preventing AR degradation (data not shown). This finding suggests that BFA may specifically activate proteasome targeting AR, supporting the notion that AR degradation is primarily mediated through BFA-activated proteasome. However, more studies are required for further confirmation and also for defining *how *BFA would actually activate proteasome.

Despite such compelling information, it cannot yet rule out the possible effect of BFA on the transcriptional level of AR (i.e. mRNA). In other words, if transcriptional activation of AR gene was somehow turned off by BFA, no AR mRNA would be transcribed, and no AR protein would be subsequently translated. This is another possible notion that the inhibition of AR transcription by BFA could lead to the inhibition of AR *de novo *synthesis. Thus, the resulting reduced level of AR protein is not due to AR degradation mediated through proteasome as described above. As it is critical to address the possible transcriptional regulation by BFA, such study is currently underway in our laboratory.

In the meantime, our separate study of BFA effect on PSA secretion (Fig. 5) may also provide useful information on understanding the mechanism of BFA-blocked androgenic action. Such a study showed that DHT-stimulated PSA secretion was completely inhibited/blocked by BFA, possibly resulting from the *preceding *inhibition of AR transcription. As PSA secretion follows its synthesis that is directly under the androgenic (DHT) regulation via "AR", the inhibition of PSA secretion indicates that no PSA gene activation (required for PSA synthesis) would have been followed because no AR transcription (required for AR synthesis) had been carried out due to the inhibition of its transcriptional activation by BFA. In other words, no AR protein would be synthesized and available for activation of PSA gene, resulting in no PSA synthesis and secretion. This may then support the notion that the inhibition of DHT-stimulated PSA secretion by BFA could mainly result from the inhibition of AR transcription.

Taken all together, BFA-induced growth inhibition appears to be primarily attributed to the greatly reduced AR protein level, although whether "it" would result from *AR degradation *(through proteasome) or *its transcriptional inhibition *remains uncertain at present. Further studies are thus warranted and required.

Nevertheless, the fact remains the same that BFA has a direct impact on AR, down-regulating its protein expression as well as its biological activity. This is indeed the primary mechanism, along with a cell cycle arrest, to induce the growth inhibition by BFA in androgen-responsive prostate cancer cells. In addition, previous studies also described the BFA-induced growth inhibition in androgen-*independent *prostate cancer cells [14,16] and primary cultures of prostatic carcinomas [15]. Therefore, these findings suggest that BFA may have the diverse growth inhibitory effects on various types of prostate cancers as well as other mammalian malignancies.

## Conclusions

In summary, BFA has a potent growth inhibitory activity, interfering with the androgen-mediated growth pathway in LNCaP cells. Specifically, BFA appears to target cell cycle and AR, inducing a G_1 _arrest and down-regulating AR activity/expression, respectively. In particular, inactivation of AR due to its degradation through BFA may primarily account for the inhibition of androgen-stimulated LNCaP cell growth. Thus, BFA could be considered a useful, effective adjuvant in ongoing androgen ablation therapy for hormone-dependent prostate cancer. Further investigations are warranted.

## Competing interests

The authors declare that they have no competing interests.

## Authors' contributions

SR is a primary investigator in charge of performing all experiments and drafting the manuscript; CA and SA serve as assistants for SR to help set up and run experiments (cell culture, cell cycle analysis, and biochemical assays); MC is the department chairman, providing us with all his support for this project; and SK is responsible for designing experiments, analyzing the data (and statistical analysis), and editing/finalizing the manuscript. All authors read and approved the final manuscript.
